# High frequency of *M. leprae* DNA detection in asymptomatic household contacts

**DOI:** 10.1186/s12879-018-3056-2

**Published:** 2018-04-02

**Authors:** Rafael Silva Gama, Thalisson Artur Ribeiro Gomides, Chaiana Fróes Magalhães Gama, Suelen Justo Maria Moreira, Fernanda Saloum de Neves Manta, Lorena Bruna P. de Oliveira, Pedro Henrique Ferreira Marçal, Euzenir Nunes Sarno, Milton Ozório Moraes, Raúl Marcel González Garcia, Lucia Alves de Oliveira Fraga

**Affiliations:** 1 Universidade Vale do Rio Doce/UNIVALE—Núcleo de Pesquisa em Imunologia, Rua Israel Pinheiro2000, B. Universitário, Governador Valadares, MG Brazil; 2 FIOCRUZ—Fundação Oswaldo Cruz, Laboratório de Hanseníase, Av. Brasil, Rio de Janeiro, RJ Brazil; 3 Universidade Federal de Juiz de Fora—Campus Governador Valadares—UFJF/GV, Rua Israel Pinheiro, 2000, B. Universitário, Governador Valadares, MG Brazil; 4 Universidade Federal de Juiz de Fora. Programa de Pós Graduação em Ciências Biológicas (Imunologia e DIP/Genética e Biotecnologia)-Rua José Lourenço Kelmer, S/n-Martelos, Juiz de Fora-MG, Brazil

**Keywords:** Leprosy, Household contacts, qPCR

## Abstract

**Background:**

Characterization of the *Mycobacterium leprae* genome has made possible the development of Polymerase Chain Reaction (PCR) systems that can amplify different genomic regions. Increased reliability and technical efficiency of quantitative PCR (qPCR) makes it a promising tool for early diagnosis of leprosy. Index cases that are multibacillary spread the bacillus silently, even before they are clinically diagnosed. Early detection and treatment could prevent transmission in endemic areas.

**Methods:**

In this study, the qPCR technique is used to detect DNA of *M. leprae* in samples of slit skin smears (SSS) of the ear lobe and blood of leprosy patients and their asymptomatic household contacts residing in Governador Valadares, MG, Brazil, a hyperendemic area for leprosy. A total of 164 subjects participated in the study: 43 index cases, 113 household contacts, and, as negative controls, 8 individuals who reported no contact with patients nor history of leprosy in the family. The qPCR was performed to amplify 16S rRNA fragments and was specifically designed for *M. leprae*.

**Results:**

Of asymptomatic household contacts, 23.89% showed bacillary DNA by qPCR in samples of SSS and blood. Also, 48.84% of patients diagnosed with leprosy were positive for qPCR while the bacillary load was positive in only 30.23% of patients. It is important to note that most patients were already receiving treatment when the collection of biological material for qPCR was performed. The level of bacillary DNA from household contacts was similar to the DNA levels detected in the group of paucibacillary patients.

**Conclusion:**

Considering that household contacts comprise a recognizable group of individuals with a high risk of disease, as they live in close proximity to a source of infection, qPCR can be used to estimate the risk of progress towards leprosy among household contacts and as a routine screening method for a chemoprophylactic protocol.

**Electronic supplementary material:**

The online version of this article (10.1186/s12879-018-3056-2) contains supplementary material, which is available to authorized users.

## Background

Leprosy is a chronic disease caused by *Mycobacterium leprae* that results in neurological and skin damage [1]. Despite advances toward the elimination of leprosy over the last two decades, new case detection rates have stabilized over the last decade, and leprosy remains an important health problem in many regions [2]. Leprosy is considered endemic in several countries with low rates of social and economic development, especially in India and Brazil, which contain the largest absolute number of cases. Given delays in the diagnosis of multibacillary (MB) leprosy, transmission of *M. leprae* from infected individuals to their contacts continues, and in many cases, irreversible nerve damage occurs before those infected are registered as patients [3, 4]. It is remarkable that DNA amplification methods have been used for genomic studies and resistance-associated gene mutations [5].

The early diagnosis and prompt initiation of treatment is essential to the rapid interruption of the disease transmission chain. In this sense, the development of a sensitive test for the diagnosis of leprosy has been one of the main objectives of research related to the disease [6]. The *M. leprae* genome sequence has made it possible to target specific sequences of the bacillus. PCR is also a sensitive technique capable of detecting 25 fg (10^− 15^ g) of *M. leprae* DNA [7, 8]. The assays have been developed for regions such as the 36-kDa [9], 18-kDa [10] or 65-kDa [11] antigens as well as repeat sequences (RLEP) and the gene encoding the 16S rRNA of *M. leprae* [12, 13]. The analysis of sensitivity and specificity of real-time quantitative PCR (qPCR) amplification of the sodA gene, 16S rRNA, RLEP, and 85BAg for the differential diagnosis of leprosy showed that the RLEP gene confers greater sensitivity (although a lower specificity) to the technique. The assay 16S rRNA, albeit less sensitive, was highly specifically suited for *M. leprae* [12]. Previous data [14] confirmed the suitability of the 16S rRNA primer for *M. leprae*, comparing it with nine other *Mycobacterium* species, including *M. tuberculosis* and *M. bovis* as well as bacteria of other genera, such as *Staphylococcus, Streptococcus*, and *Escherichia.* We screened and followed household contacts (HHCs) along with patients, testing the 16S rRNA qPCR assay to evaluate the presence of *M. leprae* DNA in patients and their asymptomatic HHCs.

## Methods

### Study group

The study was conducted in the city of Governador Valadares in eastern Minas Gerais State, Brazil. This city is considered hyperendemic for leprosy: the new case detection rate () was 1.9/10,000 people in 2015. In Minas Gerais, the rate was 0.5/10,000 in 2016, and in Brazil as a whole, it is currently around 1.2/10,000. The study’s participants included leprosy patients and HHCs who came to the Reference Center for Endemic Diseases and Special Programs (CREDEN-PES) at the Department of Public Health of Governador Valadares municipality. The index cases were diagnosed and biological samples were collected for up to three months after treatment began. All contacts were subjected to careful clinical evaluation before being considered asymptomatic. One hundred sixty-four individuals participated in the study: 43 index cases and 113 HHCs. Eight individuals who reported no contact with patients or family history of leprosy were included as a negative control (NC) group. According to the classification used in CREDEN-PES, the index cases were identified as paucibacillary (PB) or MB based on the Guidelines of the Brazilian Health Ministry [15]. PB individuals showed Tuberculoid-Tuberculoid (2), Borderline Tuberculoid (18) in its clinical form (PB, *n* = 20), and were negative in bacillary load. MB individuals showed Borderline–Borderline (3), Borderline Lepromatous (7) and Lepromatous Lepromatous (13). HHCs were grouped and assigned as follows: contacts of PB patients (HHC-PB, *n* = 52) and contacts of MB patients (HHC-MB, *n* = 61).

### Ethical approval

The study was approved by the Ethics Committee of the Universidade Vale do Rio Doce—Univale, filed under N° PQ 022/09–009. All participants signed an informed consent (IC) at the first evaluation.

### Sample collection and DNA extraction

The bacillary load technique was performed only in index cases, according to the guidelines to technical procedures: smear microscopy in leprosy [16]. The bacteriological index (BI) was calculated according to the work of Ridley and Jopling (1966) [17]. Slit skin smears (SSS) from the right earlobe and blood samples were collected for DNA extraction using the DNeasy kit (QIAGEN®). Samples of SSS stored in ethanol at 70% were thawed and centrifuged at 2000 rpm for 10 min. The extraction was performed according to protocols described by the manufacturer. The concentration of DNA in the eluate was determined by spectrophotometer (NanoDrop 1000 Spectrophotometer—Thermo Scientific). DNA extraction from blood samples followed the same procedure as mentioned above, using 50 μL of blood.

### Real time PCR assay—qPCR

The qPCR was performed using the TaqMan qPCR amplification system. The amplification target was the gene 16S rRNA specific for *M. leprae* as previously described [14]. The threshold values to define positive samples were used as described (Martinez et al., 2011; Barbieri et al., 2014), and the number of genomes was calculated by interpolating the Ct values in a dilution curve with the known number of *M. leprae* genomes using an Excel spreadsheet (Martinez et al., 2011). All qPCR reactions were run in triplicates and in the same thermocycler under calibrated conditions where positive controls with known numbers of genomes were tested (Step One, Applied Biosystems).

### Statistical analysis

Statistical analysis was performed using the GraphPad Prism version 5.0 software. The bacterial DNA levels among groups were evaluated by Mann-Whitney test and Kruskal-Wallis test (Dunn’s Multiple Comparison Test).

## Results

### Efficiency of bacillary loads and qPCR for *M. leprae* detection

Among the index cases, 25% of patients in the PB group showed *M. leprae* DNA in any sample (blood or SSS). It is important to note that all patients from the PB group had a negative BI. With respect to the MB group, it was found that 69.56% of patients were positive in both SSS or blood in qPCR (Any-Sample). On the other hand, in the MB group, the BI was positive in 56.52% of patients. It was observed that 30% of MB individuals that showed negative BI were qPCR positive. In addition, 100% of MB patients with positive BI showed a qPCR positive result. In summary, it was possible to identify *M. leprae* DNA in 48.84% of all index cases investigated while the BI was positive in only 30.23% (Table 1).

**Table 1:** Efficiency of bacilloscopy and qPCR for *M. leprae* detection

Study Group	N	Bacilloscopy	qPCR blood	qPCR SSS	blood or SSS
		N (%)	N (%)	N (%)	N (%)
PB	20	0 (0.00)	2 (10.00)	4 (20.00)	5 (25.00)
MB	23	13 (56.52)	4 (17.39)	14(60.87)	16 (69.56)
Total	43	13 (30.23)	6 (13.95)	18 (41.86)	21 (48.84)

*N* number of patients, *qPCR* Slit Skin Smear = qPCR SSS

**Fig. 1:**
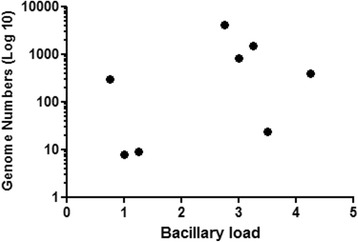
Spearman correlation between genome numbers and bacillary load for MB patients with positive bacilloscopy. (*N* = 12*; *p* = 0.3894; *r* = 0.3571) * Only individuals with bacillary DNA in SSS and positive bacilloscopy were selected

The Spearman test was used to correlate the genome numbers of *M. leprae* against the bacillary load. No association was observed (*p* = 0.3894, *r* = 0.3571) (Fig. 1). In this analysis were included only patients from the MB group who were positive for qPCR and bacillary load.

**Fig. 2:**
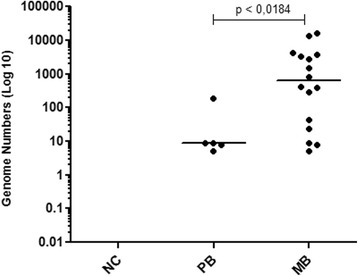
Comparison of *M. leprae* genome numbers between the groups of index cases. NC = Negative control (*n* = 8); PB = Paucibacillary (*n* = 5); MB = Multibacillary (*n* = 16). Each point represents the individual value of a genome number. The median is represented by the horizontal line. Mann-Whitney test was applied

A comparison of bacterial genome numbers between the groups of index cases was performed using interpolation of the Ct values as described above, obtained from the qPCR blood or SSS. It was observed that the genome number of *M. leprae* was significantly higher in the MB group than in the PB group (Fig. 2).

### *M. leprae* DNA detection in household contacts

The 113 HHCs were evaluated by qPCR using blood samples and SSS of the right earlobe, and it was found that 19.23% of HHC-PB were positive for *M. leprae* DNA in blood or SSS. Likewise, in relation to the HHC of MB cases (HHC-MB), 27.87% were positive. Among 113 asymptomatic contacts assessed by qPCR, 23.89% had positive *M. leprae* DNA results (Table 2).

**Table 2:** Detection of *M. leprae* DNA in household contacts of index cases

Study Group	N	qPCR blood	qPCR SSS	blood or SSS
		N (%)	N (%)	N (%)
HHC-PB	52	4 (7.69)	7 (13.46)	10 (19.23)
HHC-MB	61	7 (11.48)	11 (18.03)	17 (27.87)
Total	113	11 (9.73)	18(15.93)	27(23.89)

*N* Number of household contacts, *HHC-PB* House hold contacts Pauciballary, *HHC-MB* House hold contacts Multibacillary, *qPCR Slit Skin Smear* qPCR SSS

### Comparison of DNA levels of *M. leprae* between household contacts and groups of index cases

**Fig. 3:**
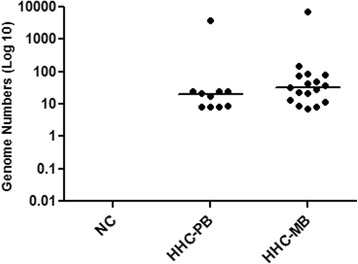
Comparison of *M. leprae* genome number among household contacts groups. NC = Negative control (n = 8); HHC-PB = Contact of paucibacillary (*n* = 10); HHC-MB = Contact of multibacillary (*n* = 17). Each point represents the individual value of a genome number. The median is represented by the horizontal line. Mann-Whitney test

**Fig. 4:**
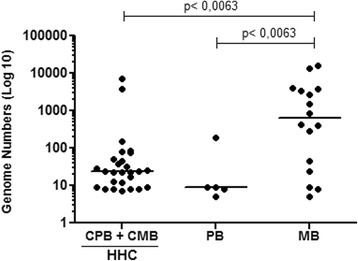
Comparison of the *M. leprae* genome number of all household contacts and index cases PB and MB. Household contacts (HHC) (*n* = 27); PB = paucibacillary (n = 5); and MB = multibacillary (n = 16). Each point represents the individual value of 1/Ct. The median is represented by the horizontal line. Kruskal-Wallis statistic and Dunn’s Multiple Comparison Test were applied

Analysis of genome numbers obtained in qPCR blood or SSS of HHCs showed no statistical difference between the HHC-PB and HHC-MB groups (Fig. 3). Interestingly, it was found that the median of the values of genome counts of all HHCs (HHC-PB and HHC-MB) was similar to the median of the index cases of PB. However, as expected, the median value of genome counts in MB cases was significantly higher than the median of the contact group (*p* < 0.0001). Note that two contacts showed similar genome numbers as the MB group (Fig. 4). After a one-year follow-up, three individuals were diagnosed with leprosy. But only one of those three infected individuals was contacted; the others moved and were not found.

## Discussion

It is clear that effective control of leprosy requires, together with multidrug therapy, new diagnostic tools that can detect *M. leprae* infection at an early stage. The PCR technique has been evaluated for DNA detection of *M. leprae* in different systems [18–20].

The high sensitivity of qPCR in relation to the BI makes this an extremely important technique in supporting the clinical diagnosis, as reported by other authors [12, 13, 20]. In our study, we confirmed the significant potential of qPCR for *M. leprae* DNA detection in leprosy cases with negative BI as well as in asymptomatic HHCs. It was observed that 25% of PB and 30% of MB individuals who showed negative BI (dimorphous 3/10) were qPCR positive. Considering that, 100% of MB patients with positive BI (4 dimorphous and 9 virchowian) were also qPCR positive (Tables 3 and 1).

**Table 3:** Characterization of the study groups for clinical form and bacilloscopy

Operational classification	Clinical form	Bacillary load	N
PB	TT	Negative	02
	BT	Negative	18
MB	BB	Negative	03
	BL	Positive	07
	LL	Positive	13
Total			43

*N* Number of patients, *PB* Paucibacillary, *MB* Multibacillary, *TT* Tuberculoid-Tuberculoid, *BT* Borderline-Tuberculoid, *BB* Borderline-Borderline, *BL* Borderline-Lepromatous, *LL* Lepromatous-Lepromatous

Although our study did not present data about the sensitivity and specificity of the qPCR test, our assay was based in a series of previous works wherein the extensive evaluation of qPCR used here is 100% specific. The sensitivity is around 50% in patients with PB forms to near 100% in patients with MB forms of the disease [21].

Because collecting SSS is an invasive procedure, it was limited to one specific site (the right earlobe). Therefore, we believe that a higher frequency of positivity for qPCR could be achieved if other collection sites could be used, as is standard for smear microscopy [16].

Importantly, some patients were already receiving treatment at the time of the collection of biological material, which may have reduced the positivity rate for qPCR, in accordance with Banerjee et al. (2010) [18].

Interestingly, we noted a moderate positive correlation (*p* = 0.047; *r* = 0.5823) between the values ​​of 1/Ct (DNA levels) and the bacterial index (BI) in MB patients, reinforcing the association between the *M. leprae* DNA level detected by qPCR and infection. Therefore, it was found that, the higher the BI, the higher the bacterial DNA level (Fig. 1) as demonstrated [22]. Evidence of a positive correlation between qPCR and BI led us to compare the *M. leprae* DNA levels among patients in an attempt to distinguish the groups. We found a significant difference in the level of bacterial DNA between the MB and PB groups (Fig. 2). Studies on leprosy transmission have demonstrated that people living with index cases are exposed to a greater risk of progressing towards the disease [23, 24]. An effective strategy for reducing the incidence of leprosy is contact tracing and diagnosis in the early stages of the disease. As shown by Banerjee et al. (2010) [18], MB contacts have increased the frequency of positivity in the multiplex PCR (M-PCR) compared to PB contacts; we also found a higher frequency of qPCR positive for HHC-MB (27.87%) in comparison with HHC-PB (19.23%). We consider that contacts of MB are more exposed to a higher bacterial load, possibly showing increased frequency of qPCR, positive in relation to HHC-PB contacts [25]. However, we did not detect significant differences in the number of genomes obtained from qPCR among HHC (HHC-PB + HHC-MB) groups.

It is important to note that all HHCs were considered asymptomatic for clinical evaluation. However, 23.89% of them had bacillary DNA in SSS of the earlobe. This high detection rate indicates a high dynamic transmission of leprosy in that study group. Knowing that leprosy has a long incubation period and that symptoms are difficult to detect in the early stages of the disease, we emphasize that the contacts’ surveillance with positive results for qPCR is extremely relevant. Six to 8% of HHCs develop clinical symptoms of leprosy within two years of diagnosis of the index case [26]. The fact that all contacts showed levels of bacterial DNA similar to the PB group can be explained by the slow progress of the disease; some of these contacts may develop leprosy in the future. In our study, with only one year of follow-up, we detected three new cases of leprosy among the examined contacts. Of these, two were CMB and one was positive for qPCR before the onset of clinical symptoms. The employment of strategy for early detection and/or identification of subclinical infection linked to chemoprophylaxis will certainly contribute to the effective control of leprosy [27]. More recently, the importance of serological and DNA-based techniques for the assessment and confirmation of diagnoses in suspected and early cases of leprosy was emphasized [28]. According Reis et al. 2014 [29], all HHC positive for qPCR should be addressed to control strategies to provide both chemoprophylaxis and immunoprophylaxis by vaccination to generate immediate protection that can be sustained in the long term.

Finally, we consider that the high sensitivity of qPCR allows the identification of a large number of asymptomatic contacts having *M. leprae* DNA. More qPCR tests need to be evaluated further as they could serve as a better diagnostic tool for early case detection and treatment to achieve faster control of leprosy.

## Conclusion

The presence of bacterial DNA in the dermal scraping earlobe suggests subclinical infection, and therefore, contacts with positive qPCR should be monitored for disease development in the future. Early detection of leprosy cases and effective chemotherapy are the best strategies to reduce the incidence of new cases of leprosy and prevent transmission. Considering that HHCs comprise a recognizable group of individuals with a high risk of disease, as they live in close proximity to a source of infection, we suggest that, as a prevention strategy, qPCR should be used to follow-up with leprosy HHCs to confirm or rule out subclinical infection.

## Additional file

Additional file 1: Bank of data. Description of data: this file contains all raw data of the study. (PDF 219 kb)

## Abbreviations

Any-Sample: Blood and/or dermal scraping
BI: Bacilloscopy index
CMB: Contacts of multibacillary
CPB: Contacts of paucibacillary
CREDEN-PES: Center for Endemic Diseases and Special Programs
Ct: Cycle threshold
DNA: Deoxyribonucleic acid
HHC: Household contact
IC: Informed consent
*M. bovis*: Mycobacterium bovis
M. leprae: Mycobacterium leprae
M. tuberculosis: Mycobacterium tuberculosis
MB: Multibacillary
M-PCR: Multiplex polymerase chain reaction
N: Number of patients
PB: Paucibacillary
PCR: Polymerase chain reaction
PGL-1: Phenolic glycolipid I
PNL: Pure neural leprosy
qPCR BL: qPCR blood
qPCR DS: qPCR dermal scraped
qPCR: quantitative polymerase chain reaction
RLEP: Repeat sequences
rRNA: Ribosomal ribonucleic acid
μL: Microliter
