# Supplementary material for: High frequency of M. leprae DNA detection in asymptomatic household contacts
Source: BMC Infect Dis. 2018 Apr 2;18:153. doi: 10.1186/s12879-018-3056-2 (PMC5879567; doi:10.1186/s12879-018-3056-2)
Supplement: Supplementary file 1 — Bank of data. Description of data: this file contains all raw data of the study. (PDF 219 kb) [file 12879_2018_3056_MOESM1_ESM.pdf]

ID	Categorization	Ct of Blood sanmple	Ct of Blood sanmple	Mean	Ct of Dermal scraped	Ct of Dermal scraped	Mean
112	CPB	Neg.	Neg.		Neg.	Neg.	
113	CPB	Neg.	Neg.		Neg.	Neg.	
137	CPB	Neg.	**35,42**	**35,42**	Neg.	Neg.	
138	CPB	Neg.	Neg.				
139	CPB	Neg.	Neg.				
148	CPB	Neg.	Neg.		Neg.	Neg.	
149	CPB	Neg.	Neg.		Neg.	Neg.	
126	CPB	Neg.	Neg.		Neg.	Neg.	
127	CPB	Neg.	Neg.		Neg.	Neg.	
146	CPB	Neg.	Neg.		Neg.	Neg.	
147	CPB	Neg.	Neg.		Neg.	Neg.	
153	CPB	Neg.	Neg.		Neg.	Neg.	
2	CPB				Neg.	Neg.	
3	CPB				Neg.	Neg.	
5	CPB				Neg.	Neg.	
10	CPB	Neg.	Neg.		Neg.	Neg.	
18	CPB	Neg.	Neg.		Neg.	Neg.	
37	CPB	Neg.	Neg.				
38	CPB	Neg.	Neg.		Neg.	Neg.	
39	CPB	Neg.	Neg.		Neg.	Neg.	
40	CPB	Neg.	Neg.		Neg.	**35,95417404**	**35,95417404**
42	CPB	Neg.	Neg.		Neg.	Neg.	
46	CPB	Neg.	Neg.		Neg.	Neg.	
47	CPB	Neg.	Neg.		Neg.	Neg.	
49	CPB	Neg.	Neg.		**35,33840179**	**35,45843506**	**35,39841843**
51	CPB	Neg.	Neg.		Neg.	Neg.	
61	CPB	Neg.	Neg.		Neg.	Neg.	

62	CPB	Neg.	Neg.		Neg.	Neg.	
63	CPB	Neg.	**37,07093**	**37,07093**	Neg.	Neg.	
64	CPB	Neg.	Neg.		Neg.	Neg.	
71	CPB	Neg.	Neg.		Neg.	Neg.	
79	CPB	Neg.	Neg.				
80	CPB	Neg.	Neg.		Neg.	Neg.	
85	CPB	Neg.	Neg.		Neg.	Neg.	
86	CPB	Neg.	Neg.		Neg.	**37,01305008**	**37,01305008**
87	CPB	Neg.	Neg.		Neg.	Neg.	
90	CPB	Neg.	Neg.		**35,34076691**	**35,75477219**	**35,54776764**
91	CPB	Neg.	Neg.		Neg.	Neg.	
93	CPB	Neg.	Neg.		Neg.	Neg.	
94	CPB	Neg.	Neg.		Neg.	Neg.	
95	CPB	Neg.	Neg.		**27,8194809**	Neg.	**27,8194809**
96	CPB	Neg.	Neg.		Neg.	Neg.	
109	CPB	**37,0754**	Neg.	**37,0754**	Neg.	Neg.	
134	CPB	Neg.	**35,34**	**35,34**	**37,62**	**38,66**	**38,14**
140	CPB	Neg.	Neg.		Neg.	Neg.	
141	CPB	Neg.	Neg.		Neg.	Neg.	
142	CPB	Neg.	Neg.		Neg.	Neg.	
143	CPB	Neg.	Neg.		Neg.	Neg.	
151	CPB	Neg.	Neg.		**36,86**	Neg.	**36,86**
152	CPB	Neg.	Neg.		Neg.	Neg.	
157	CPB	Neg.	Neg.		Neg.	Neg.	
158	CPB	Neg.	Neg.		Neg.	Neg.	
22	CMB	Neg.	Neg.		Neg.	Neg.	
23	CMB	**35,63187**	**35,29959**	**35,46573**	**37,03517914**	Neg.	**37,03517914**
31	CMB	Neg.	Neg.		**33,72317123**	Neg.	**33,72317123**
32	CMB	Neg.	Neg.		**26,92251968**	Neg.	**26,92251968**
35	CMB	**34,97571**	**34,56522**	**34,77047**	Neg.	Neg.	
36	CMB	Neg.	Neg.		Neg.	Neg.	
54	CMB	Neg.	Neg.		Neg.	Neg.	
57	CMB	Neg.	Neg.		Neg.	Neg.	
59	CMB	Neg.	Neg.		Neg.	Neg.	

60	CMB	Neg.	**36,87008**	**36,87008**	Neg.	Neg.	
81	CMB	Neg.	Neg.		Neg.	Neg.	
82	CMB	Neg.	Neg.		Neg.	Neg.	
118	CMB	Neg.	Neg.		Neg.	Neg.	
119	CMB	Neg.	Neg.				
120	CMB	Neg.	Neg.		Neg.	Neg.	
136	CMB	Neg.	**37,28**	**37,28**	Neg.	Neg.	
145	CMB	Neg.	Neg.		Neg.	Neg.	
154	CMB	Neg.	Neg.				
155	CMB	Neg.	Neg.		Neg.	Neg.	
128	CMB	Neg.	Neg.		Neg.	**35,56**	**35,56**
129	CMB	Neg.	Neg.		Neg.	Neg.	
130	CMB	Neg.	Neg.		**36,34**	Neg.	**36,34**
56	CMB				Neg.	Neg.	
28	CMB	Neg.	Neg.		Neg.	**34,9833107**	**34,9833107**
33	CMB	Neg.	Neg.		Neg.	Neg.	
75	CMB	Neg.	Neg.		Neg.	Neg.	
76	CMB	Neg.	Neg.				
98	CMB	Neg.	Neg.		Neg.	Neg.	
99	CMB	Neg.	Neg.		Neg.	**32,70974731**	**32,70974731**
100	CMB	Neg.	Neg.		Neg.	Neg.	
101	CMB	Neg.	Neg.		Neg.	Neg.	
108	CMB	Neg.	Neg.		Neg.	Neg.	
114	CMB	Neg.	Neg.		Neg.	Neg.	
115	CMB	Neg.	Neg.		Neg.	Neg.	
8	CMB	Neg.	Neg.		Neg.	Neg.	
9	CMB	Neg.	Neg.		Neg.	Neg.	
12	CMB	Neg.	Neg.		Neg.	Neg.	
13	CMB	Neg.	Neg.				
14	CMB	Neg.	Neg.		Neg.	Neg.	
15	CMB	Neg.	Neg.				
16	CMB	Neg.	Neg.		**33,52672195**	Neg.	**33,52672195**
19	CMB	Neg.	Neg.		Neg.	Neg.	
66	CMB	**36,97926**	Neg.	**36,97926**	Neg.	Neg.	

67	CMB	**34,57853**	**34,08636**	**34,33245**	Neg.	Neg.	
68	CMB	Neg.	Neg.		**34,75926971**	**34,26903915**	**34,51415253**
69	CMB	Neg.	Neg.		**36,04762268**	**34,26330566**	**35,15546417**
89	CMB	Neg.	Neg.		Neg.	Neg.	
102	CMB	Neg.	Neg.		Neg.	Neg.	
103	CMB	Neg.	Neg.		Neg.	Neg.	
104	CMB	Neg.	Neg.		Neg.	**36,48492432**	**36,48492432**
105	CMB	Neg.	Neg.		Neg.	Neg.	
106	CMB	Neg.	Neg.		Neg.	Neg.	
107	CMB	**34,06751**	**33,23258**	**33,65005**	Neg.	Neg.	
135	CMB	Neg.	Neg.		Neg.	Neg.	
144	CMB	Neg.	Neg.		Neg.	Neg.	
156	CMB	Neg.	Neg.		Neg.	Neg.	
121	CMB	Neg.	Neg.		Neg.	Neg.	
122	CMB	Neg.	Neg.		Neg.	Neg.	
123	CMB	Neg.	Neg.		Neg.	Neg.	
124	CMB	Neg.	Neg.				
125	CMB	Neg.	Neg.		Neg.	Neg.	
